# Physiological and Environmental Factors Affecting the Composition of the Ejaculate in Mosquitoes and Other Insects

**DOI:** 10.3390/insects10030074

**Published:** 2019-03-15

**Authors:** Megan E. Meuti, Sarah M. Short

**Affiliations:** Department of Entomology, The Ohio State University, 2001 Fyffe Rd., Room 232 Howlett Hall, Columbus, OH 43210, USA; short.343@osu.edu

**Keywords:** mosquito, male accessory glands, accessory gland proteins, seminal fluid, sperm, body size, larval crowding, nutrition, temperature, seasonal cues

## Abstract

In addition to transferring sperm, male mosquitoes deliver several proteins, hormones and other factors to females in their seminal fluid that inhibit remating, alter host-seeking behaviors and stimulate oviposition. Recently, bioinformatics, transcriptomics and proteomics have been used to characterize the genes transcribed in male reproductive tissues and the individual proteins that are delivered to females. Thanks to these foundational studies, we now understand the complexity of the ejaculate in several mosquito species. Building on this work, researchers have begun to identify the functions of various proteins and hormones in the male ejaculate, and how they mediate their effects on female mosquitoes. Here, we present an overview of these studies, followed by a discussion of an under-studied aspect of male reproductive physiology: the effects of biotic and abiotic factors on the composition of the ejaculate. We argue that future research in this area would improve our understanding of male reproductive biology from a physiological and ecological perspective, and that researchers may be able to leverage this information to study key components of the ejaculate. Furthermore, this work has the potential to improve mosquito control by allowing us to account for relevant factors when implementing vector control strategies involving male reproductive biology.

## 1. Introduction

Mosquitoes are by far the deadliest animals known to man, transmitting pathogens like the malaria parasite, and viruses such as yellow fever, dengue, chikungunya, Zika and West Nile which collectively claim hundreds of thousands of human lives each year [1]. Furthermore, mosquitoes threaten our pets and livestock by transmitting pathogens such as canine heartworm and the Eastern equine encephalitis virus [2,3]. Vertebrates become infected with these pathogens when an infective female mosquito takes a blood meal and transmits them from her salivary glands into the vertebrate blood. Although male mosquitoes never blood feed, and therefore do not transmit pathogens, they play a critical role in maintaining the mosquito population. Reducing the mosquito population is a primary line of defense against all of these diseases, and multiple proposed vector control methods rely on releasing sterile or genetically modified males that produce no offspring when mated with wild females [4,5,6]. It is also possible to genetically engineer mosquitoes to be more resistant to pathogens, and to propagate these traits using reproductive gene drives [7]. Due to this promising role in vector control, male mosquito reproductive biology and the ejaculate they transfer to female mosquitoes has been a subject of growing interest (see review by [8]).

**Figure 1:**
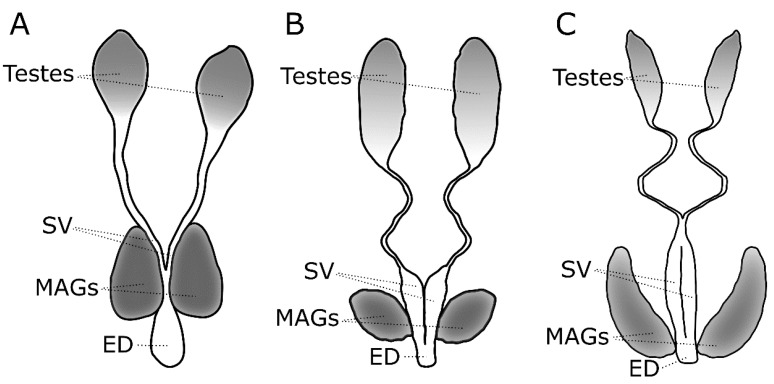
Morphology of the male reproductive tract in (**A**) *Anopheles gambiae*, (**B**) *Culex pipiens* and (**C**) *Aedes aegypti*. MAG = male accessory gland, SV = seminal vesicle, ED = ejaculatory duct.

We have long known that in addition to transferring sperm, male mosquitoes and other animals (arthropods, reptiles/amphibians, birds and mammals) transfer a variety of factors in their seminal fluid that not only activate sperm and enhance its motility and storage, but also have profound influences on female behavior, reproductive physiology and fecundity. Proteins in the seminal fluid are primarily produced in the male accessory glands and the seminal vesicles, ejaculatory duct and bulb (Figure 1). Possibly, the testes produce seminal fluid proteins as well. Collectively these seminal fluid proteins (SFPs) combined with hormones, carbohydrates, salts, and lipids make up the seminal fluid [9], and the seminal fluid along with sperm compose the male ejaculate (as reviewed in [10,11,12,13,14,15]). Decades of work primarily in the fruit fly, *Drosophila melanogaster*, have elucidated the specific effects of multiple components of the ejaculate, the molecular interactions among these components, their mode of action in the female, and the evolutionary dynamics of genes that code for SFPs (reviewed by [10,11]). This seminal work (pun intended) in a model organism has provided a critical foundation allowing further exploration of seminal fluid biology in other organisms.

Building on this substantial body of work, we are now beginning to understand the functional role of several components of the seminal fluid in mosquitoes and other insects, including how they mediate their effects on female behavior, physiology and fecundity. However, there is still much work to be done here due in large part to the complexity of seminal fluids. To date, most of the research has mainly focused on characterizing the proteins in the ejaculate, which are highly diverse. For example, a combination of bioinformatic and microarray analyses have identified 121 transcripts produced in the male accessory glands in the malaria mosquito, *Anopheles gambiae* [12,16,17], and a recent study demonstrates that males of the yellow fever mosquito, *Aedes aegypti*, transfer 870 sperm proteins and 280 SFPs to females during mating [18]. Another growing field of research has focused on the evolutionary biology of SFPs (see reviews by [14,19,20]). This work has demonstrated that although the classes of proteins found in the seminal fluids are well-conserved across mosquitoes and other insect species, there is a high degree of variation in the number and type of the individual components within these classes even between closely-related species, such as *Ae. aegypti* and *Ae. albopictus* [18,21]. Among anophelines there is evidence of at least one recent expansion of a male accessory gland specific gene family [22]. Additionally, Izquierdo et al. [23] showed that some male reproductive transcripts have consistent patterns of gene expression across the *Anopheles gambiae* species complex, but others have lineage-specific expression profiles. This suggests that genes coding for components of the ejaculate have a dynamic evolutionary history, likely due to their role in mating and reproduction (reviewed by [15,19,20]).

In addition to understanding the composition of the ejaculate and the function and evolution of its components, it is also important to understand the potential influence that mosquito physiology and the environment have on the composition of the ejaculate. We consider this to be of interest because it will not only provide a more complete understanding of mosquito reproductive biology, but it could also potentially improve the development and implementation of control strategies involving sterile or genetically modified male mosquitoes (see review by [8]). In this review, we will first discuss the characterization of accessory gland proteins and other components of the seminal fluid in mosquitoes and other insects, and the function of some of these components on female reproductive physiology. Due to the vast nature of this field, we will limit our discussion to recent advances and refer readers to previously published reviews for a comprehensive overview of the composition and function of seminal fluid [10,11,12,14,15,24,25]. Primarily, we will focus on how the composition of the ejaculate might change in response to biotic and abiotic factors including male age, size/larval crowding/food quality, the social environment that males experience, temperature, and seasonal cues. Where possible, we will highlight the physiological and environmental factors that alter the mosquito ejaculate, but we will also discuss studies done with other insects in the hope that this can inform future mosquito research.

## 2. Characterization of Mosquito Seminal Fluid Components and Their Function

Although considerably less work has been done on the role of specific ejaculatory components in mosquitoes than has been done in *D. melanogaster*, we are quickly gaining an understanding of the myriad functions of this complex mixture including how it elicits its unique effects on female mosquito reproductive physiology. Unlike most female insects which mate more than once in their lifetime (reviewed by [26]), female mosquitoes of *An. gambiae* and *Culex pipiens* mate only once [27,28] and approximately 86% of females of *Ae. aegypti* are singly mated as well [29]. Most female mosquitoes are anautogenous, requiring a blood meal in order to produce eggs, and mating can influence female host-seeking behavior [30]. The transfer of ejaculate during mating can also affect a variety of other post-copulatory phenotypes. For example, in *Ae. aegypti,* male accessory gland products have been shown to trigger increased oviposition [31,32,33,34] and refractoriness to remating in females [35,36]. Injection with male accessory gland (MAG) extract increases female blood feeding propensity [32], and increases the rate of blood digestion [37]. Transfer of male ejaculate during mating has been shown to increase female longevity in *Ae. aegypti* [38] and subsequent work revealed that this increase in longevity is elicited by male accessory gland products alone [32]. Male accessory gland products also have been shown to alter locomotor activity, making females less active during the day and increasing their activity during dusk [39]. In the Asian tiger mosquito, *Ae. albopictus*, MAG products are sufficient to induce oviposition and refractoriness to remating [36,40]. In the Northern house mosquito, *Cx. pipiens*, transplanting male accessory glands into adult females induces refractoriness [35] as does the injection of MAG homogenate into female *Cx. tarsalis* [41]. In *An. gambiae*, mating reduces a female’s receptivity to re-mating and triggers egg production [27,42]. However, spermless males also can induce these effects [43]; therefore, the inhibition of remating and stimulation of egg production are thought to primarily be due to the transfer of the hormone 20 hydroxyecdysone in the seminal fluid [44]. Additionally, the transfer of male derived 20 hydroxyecdysone also induces large scale transcriptional changes in the female reproductive tract [45]. A potential role for other male accessory gland products in these traits in *An. gambiae* remains unclear (see reviews by [12,15]), although Dottorini et al. [46] were able to suppress 50% of SFPs by using RNAi against a heat shock factor that regulates their transcription. Suppression of these SFPs did not interfere with the males’ ability to mate with females, but females mated with RNAi-treated males produced significantly fewer offspring. Lower levels of 20E also reduce sperm storage in the spermathecae [47], but the data suggest that SFPs, in addition to 20E, are likely important in mediating reproductive responses in *An. gambiae*. 

The development of molecular tools, including bioinformatic surveys of published genomes and transcriptomic studies using microarrays and RNAseq, as well as the ability to label and identify male-derived proteins, has allowed us to identify the genes that are transcribed in male accessory glands and confirm that these seminal fluid proteins and other factors are transferred to females. For example, approximately 138 male accessory gland transcripts have been identified in *An. gambiae* [12,16,46] while proteomic studies have confirmed that males of *Ae. albopictus* transfer 198 SFPs [21] and males of *Ae. aegypti* transfer 280 SFPs [18] during mating. Furthermore, Alfonso-Parra et al. [48] identified 364 differentially expressed genes in the reproductive tract of mated females of *Ae. aegypti,* and provided evidence that 60 mRNAs transcribed by the males are also transferred to females during mating. This result was further supported by Degner et al. [18] who identified an additional 46 transcripts (106 total), including 41 protein coding and 17 long, non-coding RNAs, that are putatively synthesized in the male accessory gland and transferred to females. 

We are quickly gaining a comprehensive picture of what transcripts and proteins are present in the seminal fluids of male mosquitoes, as well as in other insects. What is substantially more difficult is identifying the role of individual components of the ejaculate on reproductive processes. Progress on this front has been stymied in part because many of the seminal proteins that have been well-characterized in *D. melanogaster* lack natural homologues in mosquitoes, and because of the dearth of molecular tools available for working in mosquitoes, including RNAi stock lines. In spite of these difficulties, several groups of researchers have made considerable progress in determining the functional role of many seminal fluid components including hormones and proteins. This has been accomplished by examining protein localization within the male and female reproductive tract, characterizing interactions among the seminal fluid components, measuring how elements of the ejaculate impact female behavior and fecundity, using RNAi to knock down specific SFPs, and (when possible) comparing protein sequences to those with known functions in *Drosophila*.

### 2.1. Comparative Analysis of Seminal Proteins in Mosquitoes and Other Insects

Early work on *Ae. aegypti* identified specific proteins in the ejaculate that induced effects on female behavior. For example, Craig [35] identified a partially purified protein from the male accessory glands of *Ae. aegypti* that when injected into females caused them to be refractory to remating. This protein was later named “matrone” [49] and found to weigh between 50 kDa and 100 kDa [50]. Later, Lee and Klowden [30] identified a 7.6 kDa protein that inhibited host seeking behavior in females of *Ae. aegypti*. This has been followed by bioinformatic, microarray, transcriptomic, and proteomic analyses of the ejaculate in three medically important mosquito species: *An. gambiae*, *Ae. aegypti*, and *Ae. albopictus*. Collectively, these analyses have identified hundreds of proteins in the ejaculate [12,16,18,21,51,52]. These studies revealed that the ejaculate in mosquitoes contains several proteolysis regulators, representing nearly 14% of the total SFPs identified in *Ae. aegypti* [52] and 24% in *Ae. albopictus* [21]. These abundant proteins are likely involved in both activating sperm motility as well as possibly helping female mosquitoes process blood meals. Other abundant components of the seminal fluid include those that putatively protect sperm from oxidative stress, as well as proteins that may facilitate sperm-egg interactions, or stimulate immune responses in females [12,18,21]. Although SFPs belonging to the same functional categories are represented across mosquito species, there is little conservation of individual genes, even between closely-related species. For example, only 36.4% of the SFPs identified in the proteome of *Ae. albopictus* were conserved with *Ae. aegypti* [21], although a more recent study found that 43% of SFPs are shared between these two species [18]. Furthermore, even with ever-improving gene ontology characterizations and genome annotations, each proteomic or transcriptomic study uncovers several SFPs with unknown functions. Given the diversity and complexity of proteins in the ejaculate, characterizing each protein’s function may be unrealistic. However, with the advent of genome editing techniques such as CRISPR/Cas9, researchers can begin to determine whether the presence or absence of individual proteins affects sperm storage, fecundity, and/or female behavior.

Less is known about the proteins that are produced by sperm, but recently Degner et al. [18] used a transcriptomic analysis of the testes as well as a proteomic analysis to characterize sperm proteins in *Ae. aegypti*. Their work demonstrates that sperm proteins are more abundant and diverse than SFPs, and surprisingly, 103 putative SFPs were also found in the sperm proteome. This suggests that some SFPs may be incorporated into the sperm before the sperm are fully matured. Although it is unclear how this might occur, transcriptomic analysis also demonstrated that the testes express some SFPs at low levels [18], providing evidence that SFPs incorporated into sperm may be produced in the testes. If this is the case, the distinction between SFPs and sperm proteins is likely more nuanced. Future research will help clarify what, if any, proteins are produced by both the male accessory glands and the testes, and the role of these products in the ejaculate. 

### 2.2. Localization and Functional Characterization of Seminal Proteins within the Reproductive Tract 

Determining where components of the male ejaculate are transcribed, the dynamics of their production in males, the subtleties of their molecular interactions, and the mechanisms by which they are transferred to and stored by females will help elucidate their function. In *Ae. aegypti* and other mosquitoes, the accessory glands are composed of two cell types that are found in either anterior and posterior zones, each of which produces, stores and exports its own products [53]. Future RNAseq experiments might very well identify the specific complement of proteins that both the anterior and posterior cells of the male accessory glands produce, but as a critical first step, Alfonso-Parra et al. [54] generated transgenic mosquitoes that had the promoter of a gene coding for a previously-identified accessory gland protein, AAEL0010824, fused to enhanced green fluorescent protein. They used this construct to trace the expression pattern of the protein and found that it is expressed in the anterior cells of male accessory glands and that its expression is upregulated after mating. Using Western blotting, they confirmed that the protein is transferred to females during mating [54]. Although it is unknown whether this protein causes post-copulatory phenotypes in females, the study provided important insight into the expression dynamics of a specific SFP. Additionally, identifying this male accessory gland-specific promoter will be a useful tool for future studies of SFP function and their potential for vector control. 

So far, the functions of only a few seminal fluid proteins in mosquitoes have been uncovered, and their role is highly similar to that in other species. Sperm motility in *Culex* mosquitoes can be activated by adding trypsin, which likely acts via the MAPK pathway to activate sperm [55]. Recent work by Stephens et al. [56] identified four trypsin-like proteases that are expressed in the accessory glands of males of *Cx. quinquefasciatus*. The sequences of these four proteins were highly homologous to other trypsin-like proteases isolated from the accessory glands of males of *Ae. aegypti* and *An. gambiae*, suggesting that sperm motility might be similarly regulated by trypsin proteases across mosquito species [56]. Additionally, Rogers et al. [57] have demonstrated a critical role for transglutaminase, TG3, in cross-linking the seminal fluid protein, Plugin, to create the mating plug in *An. gambiae*. Prior to mating, TG3 is localized to anterior cells of the male accessory gland while Plugin is restricted to the posterior cells, preventing their interaction before mating, and further underscoring how the localization of specific SFPs can inform investigations on their function. Although the interaction between plugin and TG3 is mechanistically similar to how sperm coagulate in mammals, homologues of TG3 are restricted to anopheline lineages that produce a mating plug [57]. More recently, Le et al. [58] characterized the structure and activity of TG3 *in vitro*; the ultimate goal of this work is to develop chemical agents that could inhibit the action of TG3 to sterilize mosquitoes in the field.

### 2.3. Hormones and Neuropepitdes in the Seminal Fluid and Their Effects on Female Physiology and Fecundity

In addition to proteins, male mosquitoes and other insects often pass along hormones during mating, notably juvenile hormone (JH III) and ecdysteroids, that play critical roles in female reproductive physiology. After adult emergence, levels of JH increase in adult female mosquitoes, causing primary egg follicles to lengthen. Upon engorging on a blood meal, stretch receptors in the gut signal to the neurosecretory cells in the mosquito brain to produce ovarian ecdysteriogenic hormone (OEH), which in turn activates the ovaries to synthesize ecdysone. This pro-hormone is converted into its active form, 20 hydroxyecdysone (20E) by the fat body, and initiates vitellogenesis (i.e., the deposition of yolk proteins into the developing egg follicles, see review by [59]).

Males of *Ae. aegypti* transfer JH to females [60]. Clifton et al. [61] demonstrated that male-derived JH III transferred to female mosquitoes reduced previtellogenic resorption of egg follicles and increased the amount of stored ovarian lipids. Together, these effects increase the probability that an individual egg follicle will mature following a blood meal. More recently, Nouzova et al. [62] measured the relationship between nutrition, JH III titers and insemination rates in *Ae. aegypti*, as JH III itself is required for male accessory gland development and maturation. Not surprisingly, undernourished males had lower JH III titers and inseminated significantly fewer females. Clifton et al. [61] also observed that females who had mated with well-fed males laid significantly more eggs, and this was likely a result of increased JH III transfer. Recently a putative JH binding protein was discovered in the accessory gland transcriptome of the oriental fruit fly, *Bactocera dorsalis* [63], suggesting that transfer of JH from males to females during mating likely occurs outside of the Culicidae.

Males of *An. gambiae* transfer active 20E to females as a component of the mating plug [17,44] and this hormone is responsible for inducing several transcriptional, physiological and behavioral changes in the females including reducing their receptivity to remating, enhancing sperm storage and protection, and stimulating egg production [15,45,47]. It is known that 20E is responsible for inducing these changes in several anopheline species, particularly those in which males deliver a mating plug to females [44,64] and does so by inducing the expression of a gene downstream of the ecdysone receptor, namely the mating-induced stimulator of oogenesis (MISO; [44]). Recently, Thailayli et al. [65] measured short and long-term post-mating responses in field-collected females of *An. gambiae* and *An. coluzzi*, two important disease vectors that recently diverged. While the transcriptional profiles of recently mated females in both species were largely similar, they found that MISO was significantly upregulated in *An. coluzzi* females after mating, but not in recently mated *An. gambiae*, suggesting that 20E might elicit different responses within these two closely-related species.

It is possible that the different utilization of JH III in *Ae. aegypti* and 20E in *Anopheles* mosquitoes could be caused by differences in their life histories, as females of *Aedes* spp. generally take a blood meal after mating whereas females of *Anopheles* spp. frequently take a blood meal before mating (reviewed by [66]). Therefore, it may be selectively advantageous for males of *Aedes* spp. to provision females with a more preparatory hormone to induce egg follicle maturation, whereas males of *Anopheles* species may benefit from supplying females with hormones that could help her quickly convert proteins from her recently acquired blood meal into yolk proteins. However, we note that Borovsky et al. [60] demonstrated that males of *Cx. nigripalpus*, *An. rangeli*, and *An. trinkae* are also capable of synthesizing JH III in their accessory glands, suggesting that many male mosquitoes may deliver this important hormone to females during mating. Furthermore, males of several insect species including the migratory locust, *Melanoplus sanguinpes* [67] and the flour beetle, *Tribolium castaneum* [68] synthesize ecdysone in their accessory glands, and it is therefore possible that transfer of ecdysteroids may also occur broadly among insect taxa. Interestingly, Sirot et al. [52] identified a predicted sterol carrier in the Niemann-Pick type C-2 family in the seminal fluid of *Ae. aegypti*. As sterol carriers are required for ecdysone biosynthesis in *D. melanogaster* [69], this result suggests that males of *Ae. aegypti* might also synthesize ecdysone in their reproductive tissues and/or stimulate its production in females [52].

It is likely that in addition to JH and ecdysteroids, several accessory gland-produced neuropeptides are transferred to the females and that this distinct class of hormones also regulate reproductive processes. For example, Boes et al. [21] identified an adipokinetic-like hormone (AKH) in the seminal fluid of *Ae. albopicutus*, and hypothesized that it might stimulate egg production in females, consistent with its normal role in mobilizing energy reserves. Boes et al. [21] also hypothesized that AKH might play a role in protecting sperm, given previous work in the fire bug, *Pyrrhocoris apterus*, which showed that exposure to hydrogen peroxide induces AKH expression, and that co-injecting AKH and hydrogen peroxide reduces mortality [70]. Interestingly, AKH was found at a low level in the seminal fluid of *Ae. aegypti* in a 2008 study [51] but not in a more recent study [18]. Oryan et al. [71] recently demonstrated that two different isoforms of an AKH receptor are strongly expressed in the reproductive tract of females of *Ae. aegypti*. Combined, this work suggests that male-derived AKH could bind in the female reproductive tract of *Aedes* mosquitoes and might elicit important reproductive responses. Notably, Oryan et al. [71] also demonstrated the presence of a receptor for corazonin, another gonadatropin-like neuropeptide hormone, in both the male testes and the females ovaries, suggesting a role of this neuropeptide in influencing reproductive responses. 

Additional neuropeptide hormones are also synthesized in male accessory glands and have profound effects in female mosquitoes. Recently, Duvall et al. [72] discovered that males of *Ae. aegypti* transfer a short neuropeptide hormone, Head Peptide-1 (HP-1), to females and that this factor rapidly prevents females from mating with another male. Previous work demonstrated that HP-1 specifically binds to Neuropeptide Y Like Receptor 1 (NPYLR1; [73]). Duvall et al. [72] found that females that had a null mutation in the receptor, as well as wild type females that had mated with HP-1 null males, were more likely to mate multiple times and produced offspring with mixed paternity. HP-1 appears to have evolved recently in *Aedes* species and HP-1 isolated from males of *Ae. albopictus* also reduced re-mating in females of *Ae. aegypti* [72], suggesting a possible mechanism for the ability of *Ae. albopictus* males to competitively satyrize females of *Ae. aegypti* [74]. 

## 3. Physiological and Ecological Factors that Influence Seminal Fluid Composition

In addition to understanding the composition of the mosquito ejaculate and the molecular mechanisms by which it influences female physiology, it is also critical to understand the factors that influence variability in its composition. Changes in the presence/absence of a particular component or differences in the amount of a component could have dramatic implications on female reproductive biology. Understanding which biotic and/or abiotic factors alter the presence or abundance of components of the ejaculate, and how or to what extent, is critical to maximize the success of sterile insect techniques and other programs that depend on the release of genetically modified males (see review by [8]). Furthermore, it is possible that researchers could leverage differences in the composition of the ejaculate among males subjected to varying biotic and abiotic conditions to study the functions of differentially abundant seminal fluid compounds. 

### 3.1. The Effect of Lifespan/Aging on Seminal Fluid Composition

Over the course of a male insect’s lifespan, its ejaculate may change such that older males could have substantially different effects on female reproductive physiology than younger males (Table 1). For example, in males of *Ae. aegypti*, sperm number in the male reproductive tract increases dramatically early in life and then levels off later [75], and older males collected from field populations transfer more sperm at ten days post-eclosion compared to younger males [76]. In *Ae. albopictus*, sperm count increases until at least 20 days post-eclosion in large males, but in small males it increases until ten days post-eclosion and then does not increase beyond that number later in life [77]. These studies suggest that aging has clear effects on the physiology of the male mosquito reproductive tract and on sperm number, but no work has yet tested whether aging influences the composition of the seminal fluid or the abundance of SFPs in male mosquitoes. 

The results of how aging affects the ejaculate in mosquitoes is consistent with similar studies in other insect species. In the Mediterranean fruit fly, *Ceratitis capitata,* aging is positively correlated with number of sperm cells in the testes [78]. In the Mexican fruit fly, *Anastrepha ludens*, aging is negatively correlated with protein quantity in testes, although the authors did not observe any impact of this reduction in post-copulatory traits such as receptivity to remating and sperm storage in females [79]. In *D. melanogaster*, older males have more sperm and higher sperm viability relative to younger males [80], and the size of the ejaculatory bulb increases with age [81]. Additionally, as males age the expression of multiple genes that code for SFPs in the accessory glands decreases [82]. Furthermore, Leiblich et al. [83] demonstrated that older males of *D. melanogaster* that have mated multiple times transfer a subset of secretory cells from their accessory glands in their ejaculate, and that this process is dependent on the Bone Morphogenic Protein signaling pathway. As this pathway is required to prevent females from mating with other males, this suggests a mechanism by which older males can continue to induce post-mating effects in females. In *D. bipectinata*, older males transfer more protein and sperm to females compared to younger males [84]. We note in most of these studies males were aged without access to females. This was likely done to control the number of times each male mated to ensure the effects of aging were not confounded with number of copulations. However, it would be valuable to investigate whether these effects persist if male mosquitoes and other insects are given access to females and allowed to mate freely or a controlled number of times throughout their lives, especially given that the quality of the ejaculate decreases as males mate multiple times (reviewed by [14]). 

### 3.2. Body Size, Nutrition, and Crowding

Decreases in overall body size as a result of larval nutrient deprivation have been shown to impact sperm count in *Ae. aegypti* and *Ae. albopictus* (Table 1; [75,77]). In these studies, males were experimentally reared to be smaller by placing larvae in crowded and nutritionally limited conditions, and smaller males were found to produce fewer sperm than larger males. Helinski and Harrington [38] found that females mated to larger, well-fed males of *Ae. aegypti* were more fecund than those mated to smaller, nutrient-deprived males; this effect was not evident until the male had mated multiple times and may have been largely due to sperm depletion occurring more rapidly in smaller males. In another study, larger, well-fed *Ae. albopictus* males induced significantly higher fecundity in females compared to smaller, nutrient-deprived males, but this same effect was not observed in *Ae. aegypti* [85]. However, the number of times the male mosquitoes mated was not controlled, so the implications of this study for the findings of Helinski and Harrington [38] are unclear. In *D. melanogaster*, smaller males generated by larval crowding produce lower levels of two SFPs in their accessory glands, but transfer a proportionally higher amount (relative to larger males) of these SFPs to females during mating [86]. In all of these studies, the effect of male body size is confounded with that of larval diet and crowding, which could affect the ejaculate independent of their effects on male size. It may be of interest to investigate whether body size has an independent effect on the composition of the ejaculate but separating the effects of nutrition and crowding from body size may be challenging.

As adult male mosquitoes feed exclusively on nectar, it is possible that the nutritional environment they experience as larvae has a more critical influence on their ejaculate composition than that which they experience as adults. However, there is evidence that adult diet can influence the male ejaculate, suggesting that nutrition has an influence on the ejaculate independent of male body size. As previously mentioned, adult males of *Ae. aegypti* fed a low-sugar diet produce and deliver less JH III to females, and overall have lower insemination rates compared to those on a high sugar diet [61,62]. It has also been shown that male *Ae. aegypti* starved after eclosion are less effective at inducing oviposition in their mates [34] and that accessory gland homogenates from males fed on sugar were more potent at inducing refractoriness in females compared to those from males fed on canned apples [35]. Overall, these findings suggest that diet in adult male mosquitoes has the potential to influence the composition of the ejaculate in ways that can significantly influence female reproduction. 

The relationship between nutrition in adult males and reproductive phenotypes has been documented in several other species. For example, protein-deficient diets reduced sperm number in the cockroach, *Nauphoeta cinereal* [87], and female *D. melanogaster* that mated with nutritionally-deprived males became receptive to re-mating sooner than those that had mated with well-fed males [88]. However, in the latter example, it is not known whether this effect was because the nutritionally-deprived males produced lower levels of accessory gland proteins or lower amounts of sperm, or both. Xu et al. [89] demonstrated that nutritional deprivation in adulthood reduced both the growth and rate of maturation of accessory glands in the flour beetle, *Tribolium castaneum*. Similarly, Abraham et al.’s [90] work in the South American fruit fly, *Anastrepha fraterculus*, showed that accessory gland homogenates isolated from protein-fed males caused females to be refractory to remating for a longer period of time than those injected with accessory gland homogenates isolated from sugar-fed males.

These studies make it clear that food availability, larval crowding, and male size play an important role in determining the composition of the ejaculate. To begin to tease apart the role of larval nutrition independent of size, it may be beneficial to examine less extreme nutrient deprivation, or to compare diets with differing amounts of sugar, protein, and/or lipids. Differences due to size independent of nutrient deprivation could potentially be investigated by utilizing strains that differ genetically in average body size; these could be derived from natural populations or generated by artificial selection in the lab, as was done in *D. melanogaster* by Wigby and Sirot [91]. Although it may not be necessary or feasible to precisely tease apart the relationship between each of these factors *per se,* additional research on how field-relevant nutrient levels, variation in male body size, and differing larval densities affect seminal fluid composition is warranted. 

### 3.3. Social Environment of Adult Males

Another growing area of research is how the social environment, defined here as the number and interactions of adult conspecifics before and during mating, affects the amount and quality of ejaculate that is transferred to females (see review by [92]). These changes in the ejaculate are due largely to perceived differences in sperm competition and have been well-studied in *D. melanogaster*. For example, males housed with other males before mating are more likely to transfer higher amounts of multiple SFPs [91,93] and this occurs regardless of male body size [91]. In at least one study, this effect was shown to manifest early in development; larvae reared in the presence of adult males or under high density conditions (but without nutrient stress) had larger accessory glands and this was independent of any differences in body size [94]. In each of these studies, the authors hypothesized that the composition of the ejaculate changed in response to perceived sperm competition as females of *D. melanogaster*, like most other insect species, are polyandrous [26]. Additional research suggests that males of *D. melanogaster* are able to accurately assess the mating status of the female and increase the amount of sperm [95] and alter the composition of their ejaculate [96] when mating with previously inseminated females. 

Polyandry is uncommon in many mosquito species [27,28]. Therefore, whether the social environment similarly impacts the composition of the mosquito ejaculate and the extent to which sperm competition occurs is unclear. A significant proportion of *Ae. aegypti* females mate multiple times [29], and therefore determining the role of sperm competition and whether male mosquitoes alter the composition of their ejaculate in response to female mating status warrants further investigation. Beyond sperm competition, it is possible that male mosquitoes could detect heightened competition for mates due to high male density, especially for species of mosquitoes that form male swarms/mating aggregations (reviewed by [15]). Furthermore, male mosquitoes are polygynous, and therefore must balance the amount and quality of ejaculate they transfer to females to reflect the number of mating opportunities they perceive they will have in a given social environment ([86] reviews by [14,19]).

### 3.4. Temperature

The effects of temperature on the structure of the male reproductive system and/or the composition of the ejaculate has not been extensively studied in insects. Snook et al. [97] demonstrated that heat shock increases the number of sperm produced in the testes of *D. simulans*. Pedersen et al. [98] demonstrated that cold stress caused a reduction in a galactoside, [1-O-(4-O-(2-aminoethyl phosphate)-b-d-galactopyranosyl)-x-glycerol], that is primarily produced in the accessory glands of male *D. melanogaster* and is transferred to females. 

How temperature affects the composition of the seminal fluids in any mosquito species is largely unknown, but temperature can affect mosquito fecundity. Females of *Ae. krombeini* and the pitcher plant mosquito, *Wyeomia smithii*, produce more eggs when subjected to fluctuating temperatures than when reared at constant temperatures [99,100], although very large fluctuations in daily temperature decreased fecundity in *Ae. aegypti* [101]. However, temperatures between 20–30 °C did not significantly change the length of each gonotrophic cycle or alter the number of eggs produced by females of *Ae. albopictus* [102]), suggesting that perhaps temperature plays little role in fecundity and other reproductive processes in this species. Although this needs to be further explored, these studies cumulatively suggest a potential impact of temperature on fecundity. It would be valuable to investigate whether any of these differences in fecundity might in part be driven by changes in the composition of the male ejaculate. 

Several studies demonstrate that temperature can also affect a number of life history traits in male mosquitoes. For example, rearing mosquito larvae at lower, constant temperatures results in male and female mosquitoes that are larger [103] and have greater wing-length to mass ratios [104], but Mohommed and Chadee [105] found that that the size of male and female mosquitoes of *Ae. aegypti* were not significantly different when the larvae were subjected to higher or lower diurnal fluctuations in temperature. In contrast, males of *Ae. krombeini* took more time to develop when reared at constant temperatures rather than fluctuating temperatures, while females subjected to either fluctuating temperatures or constant temperatures developed at the same rate [99], suggesting that males might be more susceptible than females to temperature changes. As previously discussed, smaller males that were generated through larval crowding and nutritional deprivation produced fewer sperm than larger males [75]. It will be valuable to know whether the sperm number and other components of the ejaculate are similarly altered in males that are smaller due to temperature alterations or heat stress. 

### 3.5. Seasonal Cues

Like all organisms, insects have to precisely regulate their growth, development and reproduction to occur at appropriate times of year and thereby maximize their survival and that of their offspring. This is especially true of temperate organisms that regularly experience prolonged periods of low temperatures and limited food availability during winter. However, organisms that live in tropical and subtropical environments, including many of the most serious disease vectors, are also subjected to seasons with little rainfall. As mosquitoes depend on aquatic environments for larval development, dry seasons present unique challenges for mosquito survival. Not surprisingly, most mosquitoes and other insects have the ability to interpret and respond to seasonal cues by entering a pre-programmed developmental arrest, or diapause, that allows them to prepare for and survive inimical environmental conditions (reviewed by [106,107]). The stage at which an insect enters diapause is species-dependent, but across the Culicidae there are species that diapause as eggs (e.g., *Ae. albpoictus* [108]), larvae (e.g., *Wyeomia smithii* [109]) and adults (e.g., *An. freeborni*, [110] and *An. coluzzi* [111]). 

Notably, in most species that enter adult reproductive diapause, both males and females enter this dormant state, such that the accessory glands do not develop in diapausing males and mating does not occur until diapause has been terminated (reviewed by [112]). However, in several mosquito species, including *Culex pipiens* and *An. freeborni*, males develop their accessory glands, mate and die a few weeks later [113,114]. The females remain in a reproductive arrest after mating, failing to take a blood meal or develop their egg follicles. Instead, the females feed exclusively on nectar to accumulate fat reserves, and store and maintain the males’ sperm for the duration of winter. Given these seasonal differences in female reproductive biology, it may be selectively advantageous for males to alter the composition of their seminal fluids in response to seasonal cues. Under permissive conditions, it would be beneficial for males to transfer seminal fluid components that would stimulate host-seeking, blood meal-processing, vitellogenesis, ovulation, and oviposition. In contrast, under diapause-inducing conditions, males would benefit from down-regulating these factors and instead up-regulating antioxidants, antimicrobial proteins and/or other factors in the ejaculate that could help the female store and maintain their sperm. Presently, this is only a hypothesis, but we are actively investigating whether males of *Cx. pipiens* alter the composition of their ejaculate in response to day length. Moreover, even in mosquito species that diapause as eggs, such as *Ae. albopictus*, the paternal contribution to seasonal differences in embryonic development is also unknown. Here again, it seems possible that males could alter the composition of their ejaculate in response to seasonal cues to enhance the survival of their offspring that are preparing for diapause. 

Finally, numerous studies have demonstrated a post-diapause fitness cost to female insects, such that females that have gone through diapause as eggs, larvae, pupae, or adults produce fewer offspring than females that develop directly (reviewed by [115]). Whether or not this occurs in male mosquitoes that diapause as eggs or larvae is unknown, but if costs occur, they are likely mediated through changes in their sperm, accessory gland proteins, and/or other components of the ejaculate. Recently, Kubrak et al. [116] characterized reproductive arrest in adult males of *D. melanogaster* in response to exposure to short days and low photoperiod. This study demonstrates that while the accessory glands are significantly smaller and several immune and metabolic pathways are upregulated in dormant males, they return to the same levels as in non-diapausing flies after a 1–3 week recovery period. However, the number of sperm was significantly lower in post-diapause males, and these males copulated significantly less, fathered fewer offspring, and their offspring were less likely to survive than those of males that had not undergone diapause [116]. Given the molecular tools and the large body of knowledge of the composition and function of several factors in the ejaculate of *D. melanogaster*, future work should also characterize seasonal changes in the regulation of male accessory gland development, as well as how the ejaculate of males that have undergone reproductive arrest differs from directly-developing males. 

## 4. Conclusions

The ejaculate is a complex mixture containing hundreds of proteins, sperm, and other factors that has a profound impact on the reproductive physiology and behavior of female mosquitoes and other insects. Given the extreme diversity and complexity of these fluids, it is difficult to predict the function of a component based on its role in other species and to isolate the effects of specific proteins and other factors on female reproductive physiology. In spite of these difficulties, great progress has been made by employing powerful transcriptomic, proteomic and other molecular techniques. Therefore, we suspect it is likely only a matter of time until we understand in more detail how the ejaculate mediates its many effects in *Ae. aegypti*, *Ae. albopictus*, and *An. gambiae*, the three most important mosquito vectors of human pathogens. The characterization of the composition and function of proteins and other molecules in the seminal fluid of these species should also inform work in other mosquitoes and other insects. Given the role that these proteins and other factors play in mating competition, reproductive isolation, and sexual selection, a more comprehensive picture of the composition of seminal fluids across insects could provide insights into how new species evolve [15]. Furthermore, understanding the function of components in insect seminal fluid could lead to new developments in human and veterinary medicine. For example, discoveries about insect seminal fluid have led to improvements in in vitro fertilization and other reproductive therapies (reviewed by [117]), and Wilson et al. [118] argue that male insect accessory glands may serve as a useful model for studying the human prostate.

Although our understanding of the composition of seminal fluids in mosquitoes and other insects is increasing, we still have a very limited understanding of how physiological and environmental factors impact the quality and quantity of components in the ejaculate. However, it is becoming clearer that both abiotic and biotic factors, and their interactions, can affect the number of sperm and other components of the seminal fluid in several insect species. Nutrition, body size, and age have clear effects on the male ejaculate and consequent impacts on female reproductive physiology. It is less clear how the social environment, temperature, photoperiod and/or other seasonal indicators affect the ejaculate, but we argue that this is a ripe area that warrants further investigation. Examining how biotic and abiotic factors affect the ejaculate could allow researchers to more quickly determine the function of proteins and other components in the sperm and seminal fluid by capitalizing on their natural differences in mosquitoes reared under varied conditions. For example, it would be interesting to know whether nutritionally deprived males of *Ae. aegypti* also change the presence or reduce the amount of specific SFPs in addition to producing less JHIII. As females that mate with such undernourished males are less fecund [61,62] finding differences in a specific subset of SFPs could therefore suggest a role for these proteins in female reproductive physiology. Furthermore, with so many SFPs to characterize, this evidence could also help researchers identify top candidate genes to suppress or knock out in future experiments.

It is also essential that we understand how various physiological and environmental factors alter the composition of the ejaculate and subsequent effects on females so that we can maximize the success of our control efforts by releasing genetically modified and/or sterilized male mosquitoes that can out-compete untreated conspecifics. To do so, we must first understand how the ejaculate changes in response to field-relevant stresses. Then we will be able to determine how to rear or engineer male mosquitoes in the lab to optimize the composition of their ejaculate. Several important studies have already characterized the genes expressed in male accessory glands and identified the SFPs and sperm proteins that are transferred to females. This has laid the groundwork for this exciting area of investigation and will greatly facilitate analyses of how physiological and environmental factors can impact the male ejaculate.

## Figures and Tables

**Table 1:** The effects of biotic and abiotic factors on the ejaculate of various insect species.

Physiological or Environmental Factor	Species	Effect on Ejaculate	Reference(s)
Aging	*Ae. aegypti*	Increase sperm number in testes and sperm transfer to females *	[75,76]
	*Ae. albopictus*	Increase sperm number in testes *	[77]
	*Ceratitis capitata*	Increase sperm number in testes *	[78]
	*Anastrepha ludens*	Decrease protein quantity in testes *	[79]
	*D. melanogaster*	Increase sperm number in the reproductive tract, larger ejaculatory ducts, reduces SFP expression *	[80,81,82]
	*D. bipectinata*	Increases in sperm transfer and proteins *	[84]
Small body size/poor larval nutrition/larval crowding	*Ae. aegypti*	Produce fewer sperm, and induce lower female fecundity if the males had mated multiple times	[38,75]
	*Ae. albopictus*	Produce fewer sperm, and induce lower female fecundity	[75,85]
	*D. melanogaster*	Produce lower levels of sex peptide & ovulin in accessory glands but transfer a higher proportion to females	[86]
Poor adult diet	*Ae. aegypti*	Produce less JH, have lower insemination rates, induce less oviposition, and lower refractoriness to remating	[34,35,61,62]
	*Nauphoeta cinereal*	Reduces sperm number	[87]
	*T. castaneum*	Reduces growth and maturation of accessory glands	[89]
Perceived mating competition	Mosquitoes	**Unknown**	
	*D. melanogaster*	Transfer higher amounts of several SFPs and, if exposed to males as larvae, develop larger accessory glands	[91,93,94]
Mating status of the female	Mosquitoes	**Unknown**	
	*D. melanogaster*	Deliver more sperm, and less ovulin to females that had previously mated, but similar amounts of sex peptide	[95,96]
Temperature fluctuations and/or heat stress	Mosquitoes	**Unknown**	
	*D. simulans*	Increase number of sperm in testes	[97]
	*D. melanostaster*	Reduce the amount of a galactoside that is produced in accessory glands and transferred to females	[98]
Seasonal cues	Mosquitoes	**Unknown**	
	*D. melanogaster*	Decrease sperm number in males that had undergone diapause	[116]

* Older males were virgins; it is unclear whether allowing them to mate multiple times would yield the same results.
